# Impact of type 2 diabetes and the metabolic syndrome on myocardial structure and microvasculature of men with coronary artery disease

**DOI:** 10.1186/1475-2840-10-80

**Published:** 2011-09-19

**Authors:** Duncan J Campbell, Jithendra B Somaratne, Alicia J Jenkins, David L Prior, Michael Yii, James F Kenny, Andrew E Newcomb, Casper G Schalkwijk, Mary J Black, Darren J Kelly

**Affiliations:** 1 Department of Molecular Cardiology, St. Vincent's Institute of Medical Research, Fitzroy, Australia; 2 Department of Medicine, University of Melbourne, St. Vincent's Health, Fitzroy, Australia; 3 Department of Surgery, University of Melbourne, St. Vincent's Health, Fitzroy, Australia; 4 Department of Cardiology, St. Vincent's Health, Fitzroy, Australia; 5 Department of Cardiothoracic Surgery, St. Vincent's Health, Fitzroy, Australia; 6 Department of Internal Medicine, University of Maastricht, Maastricht, The Netherlands; 7 Department of Anatomy and Developmental Biology, Monash University, Clayton, Australia

**Keywords:** Diabetic cardiomyopathy, type 2 diabetes, metabolic syndrome, fibrosis, capillary length density, advanced glycation end-products

## Abstract

**Background:**

Type 2 diabetes and the metabolic syndrome are associated with impaired diastolic function and increased heart failure risk. Animal models and autopsy studies of diabetic patients implicate myocardial fibrosis, cardiomyocyte hypertrophy, altered myocardial microvascular structure and advanced glycation end-products (AGEs) in the pathogenesis of diabetic cardiomyopathy. We investigated whether type 2 diabetes and the metabolic syndrome are associated with altered myocardial structure, microvasculature, and expression of AGEs and receptor for AGEs (RAGE) in men with coronary artery disease.

**Methods:**

We performed histological analysis of left ventricular biopsies from 13 control, 10 diabetic and 23 metabolic syndrome men undergoing coronary artery bypass graft surgery who did not have heart failure or atrial fibrillation, had not received loop diuretic therapy, and did not have evidence of previous myocardial infarction.

**Results:**

All three patient groups had similar extent of coronary artery disease and clinical characteristics, apart from differences in metabolic parameters. Diabetic and metabolic syndrome patients had higher pulmonary capillary wedge pressure than controls, and diabetic patients had reduced mitral diastolic peak velocity of the septal mitral annulus (E'), consistent with impaired diastolic function. Neither diabetic nor metabolic syndrome patients had increased myocardial interstitial fibrosis (picrosirius red), or increased immunostaining for collagen I and III, the AGE Nε-(carboxymethyl)lysine, or RAGE. Cardiomyocyte width, capillary length density, diffusion radius, and arteriolar dimensions did not differ between the three patient groups, whereas diabetic and metabolic syndrome patients had reduced perivascular fibrosis.

**Conclusions:**

Impaired diastolic function of type 2 diabetic and metabolic syndrome patients was not dependent on increased myocardial fibrosis, cardiomyocyte hypertrophy, alteration of the myocardial microvascular structure, or increased myocardial expression of Nε-(carboxymethyl)lysine or RAGE. These findings suggest that the increased myocardial fibrosis and AGE expression, cardiomyocyte hypertrophy, and altered microvasculature structure described in diabetic heart disease were a consequence, rather than an initiating cause, of cardiac dysfunction.

## Background

Type 2 diabetes and the metabolic syndrome (MetS) are associated with impaired diastolic function and an increased risk of heart failure [1,2]. There is increasing evidence for a specific diabetic cardiomyopathy, independent of coronary artery disease and hypertension, and a similar mechanism may account for the increased heart failure risk associated with the MetS [1-6]. Myocardial fibrosis and cardiomyocyte hypertrophy are the most frequently proposed mechanisms for the impaired diastolic function of diabetes, and morphological changes in small vessels of the diabetic myocardium and reduced capillary length density have also been described [1,2,5]. In addition, advanced glycation end-products (AGEs) are proposed to contribute to diabetic cardiomyopathy by cross-linking myocardial proteins such as collagen and elastin, and by promoting collagen accumulation [7]. Evidence for these mechanisms comes mainly from rodent models of diabetes [7-11], and the increased myocardial fibrosis in rodent models has led to the development of genetic models to examine its pathogenesis and to test antifibrotic therapies [8,9]. There is, however, uncertainty about the role of these mechanisms in the human diabetic heart. There are reports of increased interstitial fibrosis and collagen deposition [12-16], and reports of no difference in fibrosis between diabetic and non-diabetic hearts [17-19], although the impact of diabetes on fibrosis may depend on concomitant hypertension [18]. A key limitation of previous studies of the human diabetic heart is that many were small autopsy studies of end-stage disease that did not allow separation of the effects of diabetes from those of co-morbidities including heart failure and renal disease [3,12,14,16,18,20]. Non-autopsy studies included patients with impaired left ventricular (LV) function and heart failure [17,21-23], and there is uncertainty whether the changes observed were the cause or the consequence of impaired cardiac function.

The present study was undertaken to investigate the association of type 2 diabetes and the MetS with myocardial fibrosis, cardiomyocyte size, capillary length density, diffusion radius, arteriolar dimensions, and myocardial expression of the advanced glycation end-product (AGE) Nε-(carboxymethyl)lysine (CML) and the receptor for AGEs (RAGE). We obtained LV biopsies from patients without heart failure or previous myocardial infarction who were undergoing coronary artery bypass graft surgery. By comparing MetS (pre-diabetic) and diabetic patients with control patients without these conditions, we examined the effects of the insulin-resistant state before diabetes (and anti-diabetes medication) commenced, and the effects of diabetes before heart failure developed. Although we obtained LV biopsies from both men and women, preliminary analysis showed gender-specific differences in myocardial structure [24]; therefore, given the smaller number of women recruited to this study, the present analysis was confined to men.

## Methods

### Patients

Details of the Cardiac Tissue Bank have been previously described [24]. The St. Vincent's Health Human Research Ethics Committee approved this research and all patients gave written informed consent. From the Tissue Bank we selected all of 46 male patients having coronary artery bypass graft surgery alone, who did not have heart failure or atrial fibrillation, had not received loop diuretic therapy, and did not have evidence of previous myocardial infarction. Absence of previous myocardial infarction was established from the clinical history, electrocardiogram and troponin measurements, and was confirmed by inspection of the ventriculogram, transthoracic and transesophageal echocardiography, and examination of the heart at surgery. All patients had normal or near-normal LV systolic function as assessed by pre-operative transthoracic echocardiography and/or ventriculogram, with LV ejection fraction ≥ 50%. Intra-operative hemodynamics were measured after induction of anesthesia. A partial-thickness wedge-shaped biopsy was taken during surgery, immediately after cardioplegia, from a region of the lateral wall of the LV near the base of the heart, between the territories of the left anterior descending and circumflex arteries, that was free of any macroscopic pathology, without evidence of ischemia or wall motion abnormality on pre-operative or intra-operative imaging studies, as previously described [24].

Of the 46 patients, 13 control patients had neither MetS nor diabetes, 10 had type 2 diabetes, and 23 had MetS. MetS was defined according to the International Diabetes Federation [25]. For patients in whom abdominal circumference was not measured, based on the relationship between abdominal circumference and BMI [26], those with BMI > 25 kg/m^2 ^were considered to exceed the abdominal circumference threshold for MetS. A patient had diabetes if a history of diabetes was evident from use of glucose-lowering medications and/or insulin or if fasting plasma glucose was ≥ 7 mmol/L [27]. Of the 10 patients with diabetes, two were newly diagnosed and treated with diet alone, three were treated with insulin alone, two with insulin and metformin, two with metformin and gliclazide, and one with gliclazide alone. The mean duration of diabetes was 12 (range 0-30) years and the mean HbA1 c was 7.3% (range 5.3-9.8%, n = 7). HbA1 c was not measured routinely as part of patient enrolment in the Tissue Bank, and 3 patients did not have recent HbA1 c measurement before surgery.

### Biochemistry

Blood Hb and HbA1c, and plasma levels of creatinine were measured as part of the routine pre-surgery workup. All other variables were measured on fasting blood collected on the day of surgery, before induction of anesthesia. Blood Hb and HbA1c, and plasma levels of glucose, insulin, lipids, and creatinine were measured by St. Vincent's Health Pathology using routine clinical methods. Estimated glomerular filtration rate (eGFR) was calculated from the Modification of Diet in Renal Disease formula [28]. Insulin resistance (HOMA2-IR), insulin sensitivity (HOMA2-%S), and β-cell function (HOMA2-%B), were calculated using the HOMA calculator version 2.2 [29]. CML was measured by ELISA (Microcoat, Penzberg, Germany). Low molecular weight fluorophores (LMWF) were measured by fluorescence spectroscopy [30]. Soluble RAGE was measured by ELISA (R&D Systems Inc., Minneapolis, MN). Amino-terminal-pro-B-type natriuretic peptide (NT-proBNP) was measured by electrochemiluminescence immunoassay using an Elecsys instrument (Roche Diagnostics, Basel, Switzerland).

### Histological analysis

Details of tissue collection and fixation have been previously described [24]. All histological analyses were performed blind to patient identity and group allocation. Picrosirius red-stained 4 μm paraffin sections were analyzed for interstitial and perivascular fibrosis and arteriolar dimensions by quantitative morphometry of digitized images of the whole myocardial section (Aperio Technologies, Inc., CA). Myocardial total fibrosis was calculated using the positive pixel count algorithm as the area of collagen staining expressed as a percentage of the total myocardial tissue area, after excluding the pericardium, whereas interstitial fibrosis was calculated as described for total fibrosis, with exclusion of perivascular fibrosis.

Perivascular fibrosis was calculated as the ratio of the area of perivascular fibrosis to the total vessel area (area of vessel wall plus lumen) as determined by planimetry [31], for arterioles with mean diameter (average of maximum and minimum diameter of each arteriole) of 39 μm (range 12-151 μm). Arteriolar wall area/circumference ratio was measured for arterioles with mean average diameters of 20-80 μm. Cardiomyocyte width, determined on 4 μm sections of paraffin-embedded tissue stained for reticulin [32], was the mean of > 100 measurements for each section of the shortest diameter of cardiomyocyte profiles containing a nucleus. Measurement of capillary length density, which is the length of capillaries per unit volume of tissue, has been previously described [24].

Immunohistochemistry for collagen I and collagen III was performed in frozen sections using mouse monoclonal antibodies ab6308 and ab6310 (Abcam, Cambridge, UK), respectively. Myocardial total collagen I and collagen III densities were calculated using the positive pixel count algorithm (Aperio Technologies, Inc., CA) as the area of collagen staining expressed as a percentage of the total myocardial tissue area, after excluding the pericardium. Immunohistochemistry for CML was performed in paraffin sections using a mouse monoclonal antibody as described by Schalkwijk et al. [33]. Immunohistochemistry for RAGE was performed with a goat polyclonal antibody AB5484 (Millipore, Billerica, MA). Immunostaining of arteriolar media and intima for CML and of arteriolar intima and capillaries for RAGE was individually scored by its intensity as 0+, 1+, 2+, or 3+, after inspection of the digitized image of the whole of each section.

### Statistical analysis

The significance of differences between study groups was determined by analysis of variance (ANOVA) for continuous variables and Fisher's exact test for categorical variables. Continuous data were logarithmically transformed when necessary to normalize variances. The Fisher's Protected Least Significant Difference test and the Bonferroni correction were used for multiple comparisons of continuous and categorical variables, respectively. Correlations were estimated using Pearson correlation coefficients. All tests were two-tailed. Differences were considered significant at *P *< 0.05.

## Results

### Patient characteristics

Patient characteristics are shown in Table 1. The three patient groups were of mean age 63-66 years, with similar extent of coronary artery disease, occluded coronary arteries, collaterals, and numbers of coronary grafts performed. Diabetic patients had increased plasma glucose levels and both MetS and diabetic patients had increased BMI, plasma triglyceride levels, fasting plasma insulin, reduced insulin sensitivity and increased insulin resistance. Diabetic patients also had increased plasma creatinine and reduced eGFR. The three patient groups otherwise had similar clinical and biochemical characteristics, with similar plasma C-reactive protein, NT-proBNP, CML, LMWF, and soluble RAGE levels, and received similar therapies, apart from anti-diabetic therapies.

**Table 1:** Characteristics of control, metabolic syndrome, and diabetic men undergoing coronary artery bypass graft surgery

Parameter	Control	Metabolic syndrome	Diabetic
n	13	23	10

Age, years	66 ± 2	63 ± 2	66 ± 3

Left main stenosis > 50%, n (%)	6 (46%)	15 (65%)	3 (30%)

One vessel stenosis > 70%, n (%)	3 (23%)	6 (26%)	1 (10%)

Two vessel stenosis > 70%, n (%)	7 (54%)	11 (48%)	6 (60%)

Three vessel stenosis > 70%, n (%)	3 (23%)	5 (22%)	3 (30%)

Patients with occluded coronary artery, n (%)	5 (38%)	7 (30%)	5 (50%)

Coronary collaterals, Rentrop grade 2 or 3, n (%)	5 (38%)	12 (52%)	5 (50%)

Previous percutaneous transluminal coronary angioplasty, n (%)	2 (15%)	4 (17%)	1 (10%)

Wall motion abnormality, n (%)	2 (15%)	2 (9%)	1 (10%)

Coronary grafts/patient, n	3.4 ± 0.3	3.4 ± 0.2	3.6 ± 0.2

BMI (kg/m^2^)	25.3 ± 0.8	30.1 ± 0.7*	30.2 ± 1.3†

BSA (m^2^)	1.93 ± 0.05	2.06 ± 0.03†	2.05 ± 0.06

Clinical risk factors			

Pre-admission systolic blood pressure (mmHg)	127 ± 3	134 ± 3	133 ± 4

Pre-admission diastolic blood pressure (mmHg)	74 ± 2	76 ± 2	77 ± 3

Previous hypertension, n (%)	7 (54%)	20 (87%)	8 (80%)

Ever smoked, n (%)	7 (54%)	15 (65%)	6 (60%)

Fasting plasma total cholesterol (mmol/L)	3.5 ± 0.2	3.7 ± 0.3	3.1 ± 0.2

Fasting plasma LDL cholesterol (mmol/L)	2.1 ± 0.2	2.2 ± 0.2	1.7 ± 0.2

Fasting plasma HDL cholesterol (mmol/L)	1.03 ± 0.04	0.93 ± 0.05	0.88 ± 0.06

Fasting plasma triglyceride (mmol/L)	1.08 ± 0.04	2.02 ± 0.21*	1.86 ± 0.26†

Fasting plasma glucose (mmol/L)	5.6 ± 0.2	5.9 ± 0.1	8.1 ± 0.5‡,§

Fasting plasma insulin (pmol/L)	45 ± 11	84 ± 11*	149 ± 53‡

β cell function from HOMA2-%B	65 ± 11	92 ± 9	67 ± 11

Insulin sensitivity from HOMA2-%S	167 ± 22	90 ± 11‡	65 ± 14‡

Insulin resistance from HOMA2-IR	0.8 ± 0.2	1.5 ± 0.2*	2.5 ± 0.7‡

Plasma CML (μmol/L)	2.0 ± 0.2	2.2 ± 0.1	2.2 ± 0.1

Plasma LMWF (AU/mL)	2.6 ± 0.2	2.6 ± 0.2	2.8 ± 0.3

Plasma soluble RAGE (pg/mL)	604 ± 96	642 ± 60	753 ± 108

Plasma NT-proBNP (pmol/L)	16 ± 4	14 ± 2	24 ± 6

Hb (g/L)	14.4 ± 0.3	14.8 ± 0.3	13.3 ± 0.6||

Plasma creatinine (μmol/L)	91 ± 4	91 ± 4	105 ± 4†,||

eGFR (mL/min per 1.73 m^2^)	74 ± 4	76 ± 3	63 ± 3†,||

C-reactive protein (mg/L)	2.7 ± 0.9	5.5 ± 2.2	3.6 ± 1.2

Medications			

ACE inhibitor therapy, n (%)	5 (38%)	11 (48%)	8 (80%)

ARB therapy, n (%)	2 (15%)	8 (35%)	1 (10%)

ACEI and/or ARB therapy, (%)	7 (54%)	18 (78%)	9 (90%)

Statin therapy, n (%)	11 (85%)	20 (87%)	9 (90%)

Aspirin therapy, n (%)	7 (54%)	14 (61%)	5 (50%)

Calcium antagonist therapy, n (%)	2 (15%)	6 (26%)	2 (20%)

β-blocker therapy, n (%)	11 (85%)	15 (65%)	7 (70%)

Long-acting nitrate therapy, n (%)	1 (8%)	4 (17%)	5 (50%)

Thiazide or indapamide therapy, n (%)	3 (23%)	4 (17%)	3 (30%)

Data shown as means ± SEM or n (%). *p < 0.01; †*p *< 0.05; ‡*p *< 0.001 in comparison with control; §*p *< 0.001; ||*p *< 0.05 in comparison with metabolic syndrome. One metabolic syndrome patient with left main stenosis > 50% did not have other vessel stenosis > 70%. Coronary collaterals were scored according to Rentrop et al. [48]. ARB, angiotensin receptor blocker; CML, Nε-(carboxymethyl)lysine; eGFR, estimated glomerular filtration rate calculated using the Modification of Diet in Renal Disease study equation [28]; HOMA, Homeostasis Model Assessment calculator version 2.2 [29]; LMWF, low molecular weight fluorophore; NT-proBNP, amino-terminal-pro-B-type natriuretic peptide; RAGE, receptor for advanced glycation end-products.

### Hemodynamics and echocardiography

**Figure 1:**
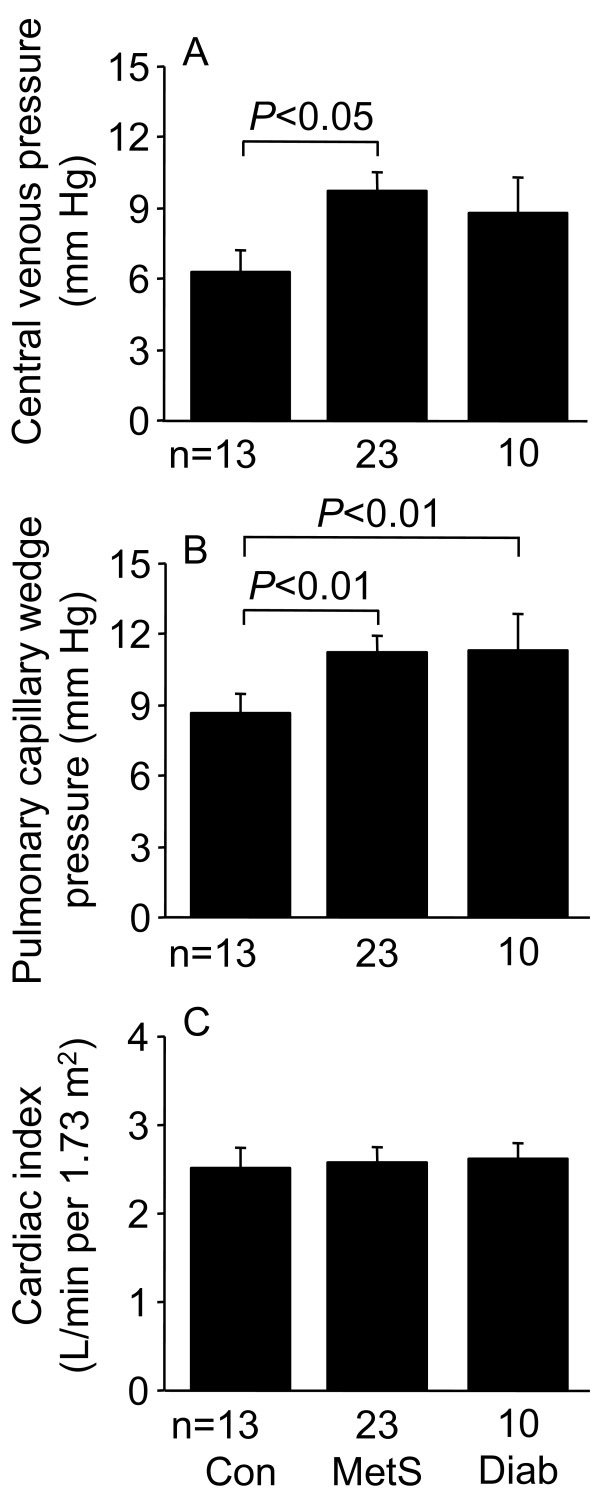
**Central venous pressure (A), pulmonary capillary wedge pressure (B), and cardiac index (C) in control (Con), metabolic syndrome (MetS), and type 2 diabetic (Diab) men undergoing coronary artery bypass graft surgery. **Data shown as means ± SEM. Measurements were made immediately after induction of anesthesia. Pulmonary capillary wedge pressure data were analyzed with central venous pressure as a covariate because of its dependence on central venous pressure.

**Figure 2:**
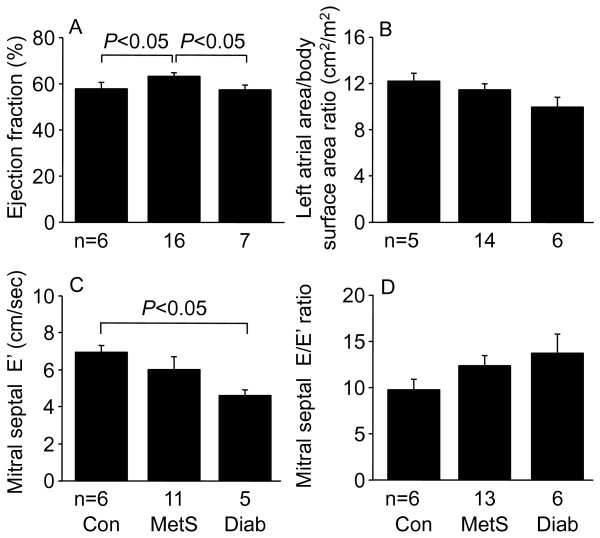
**Ejection fraction (A), left atrial area/body surface area ratio (B), early diastolic peak velocity of the septal mitral annulus, E' (C), and the mitral Doppler flow velocity E wave/E' ratio (D) in control (Con), metabolic syndrome (MetS), and type 2 diabetic (Diab) men undergoing coronary artery bypass graft surgery**. Data shown as means ± SEM. Measurements were made by transthoracic echocardiography before surgery.

Whereas cardiac index was not different between the three patient groups, MetS patients had higher central venous pressure than control patients. Because of the close correlation between central venous pressure and pulmonary capillary wedge pressure (r = 0.80, P < 0.0001), pulmonary capillary wedge pressure data were analyzed with central venous pressure as a covariate, and both MetS and diabetic patients had higher pulmonary capillary wedge pressures than control patients, consistent with impaired diastolic function (Figure 1). Pre-operative transthoracic echocardiography, performed by either the referring institution or by St. Vincent's Health, was not performed in all patients. MetS patients had slightly higher LV ejection fraction than control and diabetic patients (Figure 2). Control, MetS, and diabetic patients had similar left atrial area/body surface area ratio, but diabetic patients had reduced mitral diastolic peak velocity of the septal mitral annulus, E', also consistent with impaired diastolic function. Although the higher mitral Doppler flow velocity E wave/E' ratio of MetS and diabetic patients did not achieve statistical significance, the E/E' ratio for the three patient groups combined correlated with pulmonary capillary wedge pressure (r = 0.44, P = 0.03). There were no differences between the three patient groups with respect to mitral Doppler flow velocity E and A waves, E/A ratio, or mitral deceleration time (data not shown).

### Total, interstitial and perivascular fibrosis

**Figure 3:**
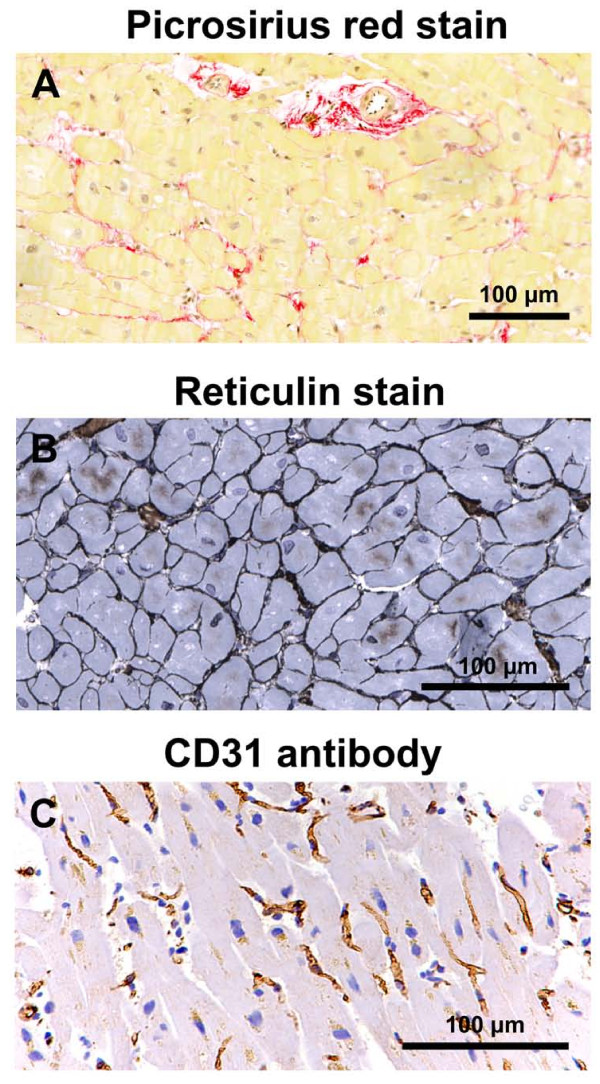
**Picrosirius-red staining demonstrating interstitial and perivascular fibrosis (stained red) and arteriolar dimensions (A), reticulin staining of cardiomyocyte membranes demonstrating cardiomyocyte size (B), and CD31 staining of capillaries (C) of left ventricular biopsies from control coronary artery bypass graft surgery patients**.

**Figure 4:**
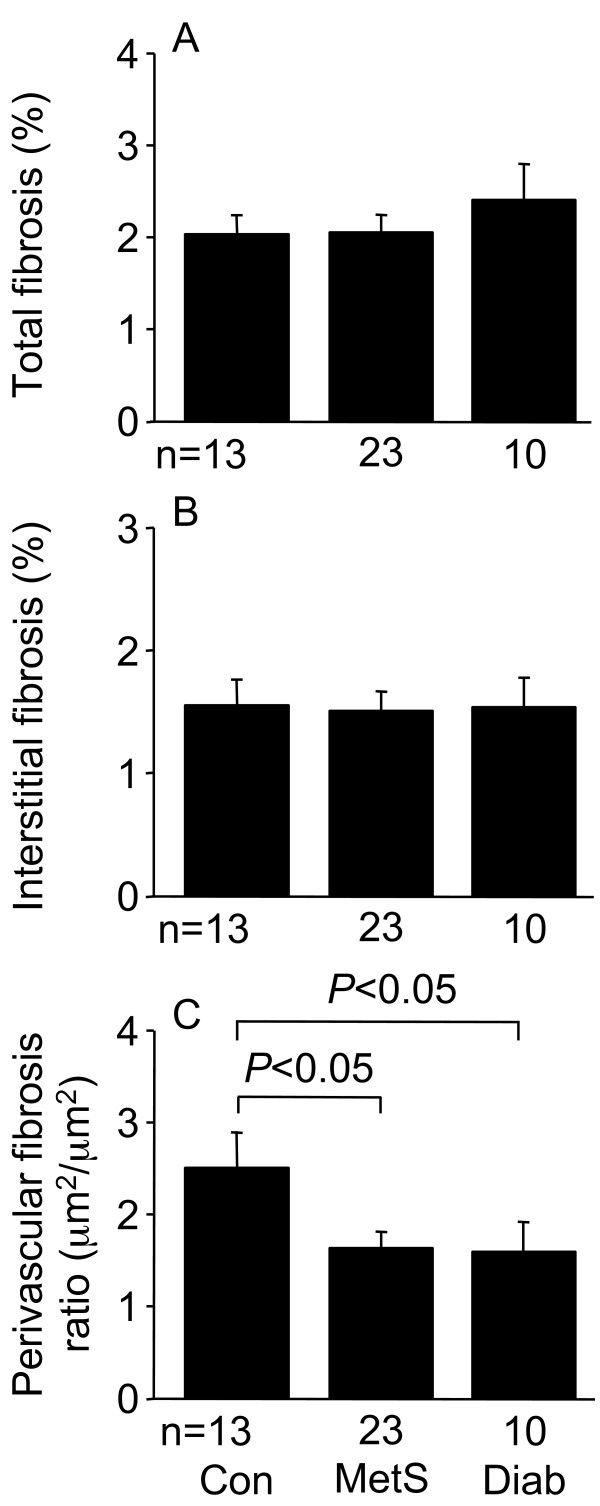
**Total myocardial fibrosis (A), interstitial fibrosis (B), and perivascular fibrosis (C) determined by picrosirius red staining in left ventricular biopsies from control (Con), metabolic syndrome (MetS), and type 2 diabetic (Diab) men undergoing coronary artery bypass graft surgery**. Data shown as means ± SEM.

**Figure 5:**
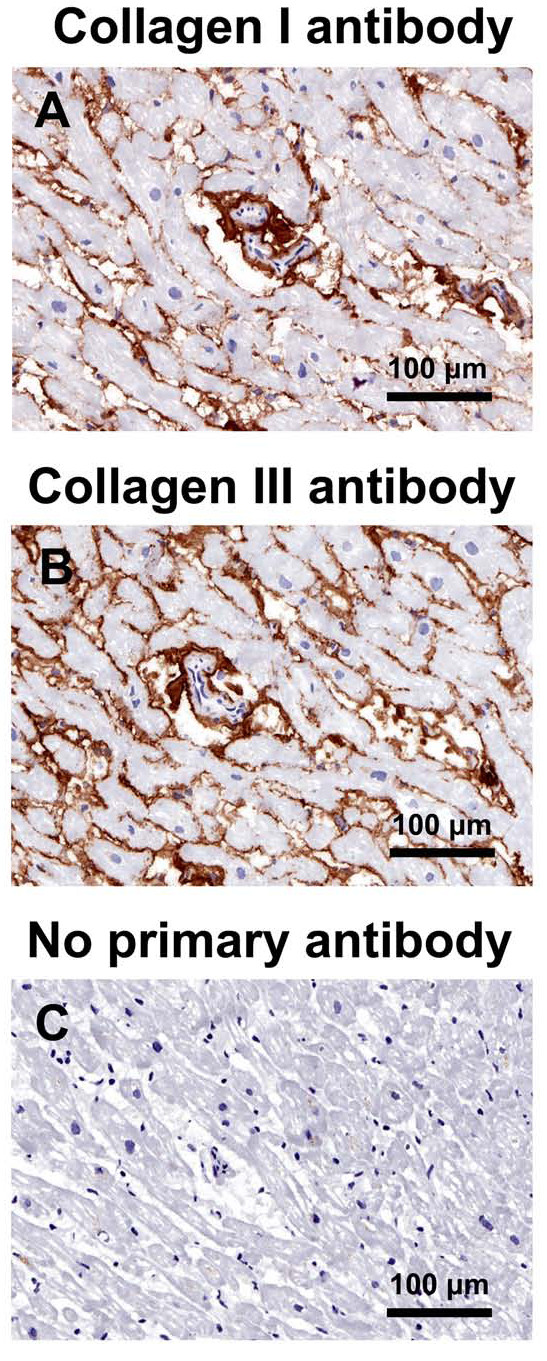
**Immunostaining for collagen I (A), collagen III (B), and a negative control section without primary antibody (C), of a left ventricular biopsy from a control coronary artery bypass graft surgery patient**.

**Figure 6:**
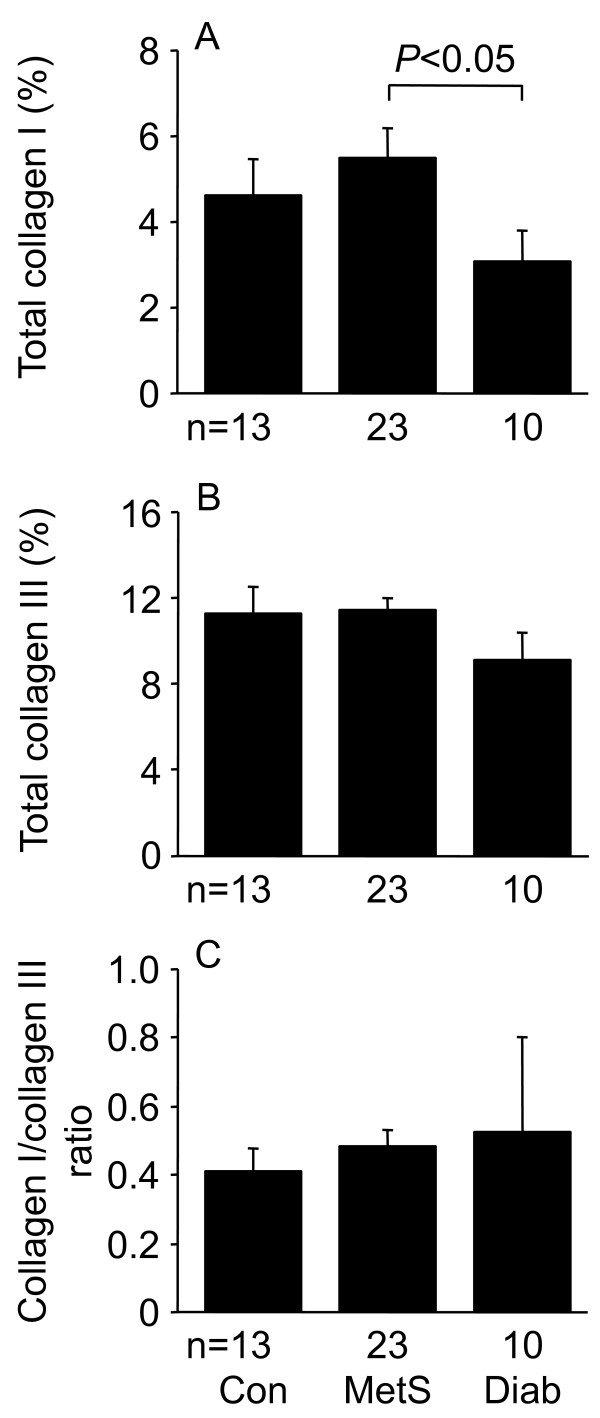
**Total collagen I (A), total collagen III (B), and total collagen I/collagen III ratio (C) determined by immunostaining in left ventricular biopsies from control (Con), metabolic syndrome (MetS), and type 2 diabetic (Diab) men undergoing coronary artery bypass graft surgery**. Data shown as means ± SEM.

The mean area of myocardial paraffin sections (3.6-4.8 mm^2^) was similar for the different patient groups. Total and interstitial fibrosis were similar for the three patient groups, whereas perivascular fibrosis of MetS and diabetic patients was less than in control patients (Figures 3, 4). Total collagen I of diabetic patients was less than for MetS patients, whereas collagen III and the collagen I/collagen III ratio, as assessed by immunohistochemistry, were similar for the three patient groups (Figures 5, 6).

### Cardiomyocyte width, capillary length density, and arteriolar dimensions

**Figure 7:**
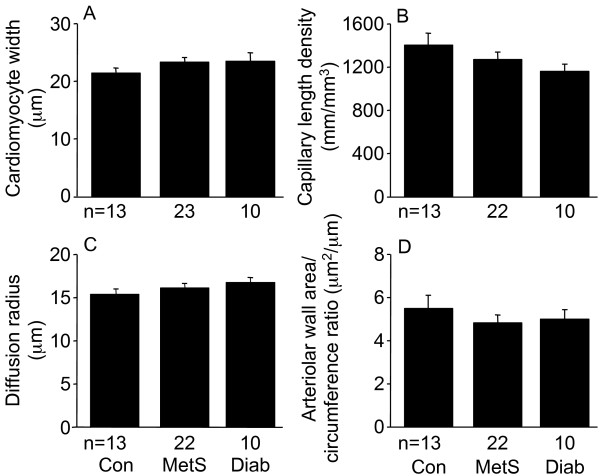
**Cardiomyocyte width (A), capillary length density (B), diffusion radius (C), and arteriolar wall area/circumference ratio (D) in left ventricular biopsies from control (Con), metabolic syndrome (MetS), and type 2 diabetic (Diab) men undergoing coronary artery bypass graft surgery**. Arteriolar wall area/circumference ratio was determined for arterioles with diameter (average of maximum and minimum diameter of each arteriole) of 20-80 μm. Data shown as means ± SEM.

Cardiomyocyte width, capillary length density, and diffusion radius did not differ between control, MetS and diabetic patients (Figures 3, 7). Arteriolar wall area/circumference ratio was measured for arterioles with diameters (average of maximum and minimum diameters for each arteriole) of 20-80 μm; mean average arteriolar diameters were 36-37 μm. There were no differences between the three patient groups in arteriolar wall area/circumference ratio (Figure 7).

### Immunostaining for CML and RAGE

**Figure 8:**
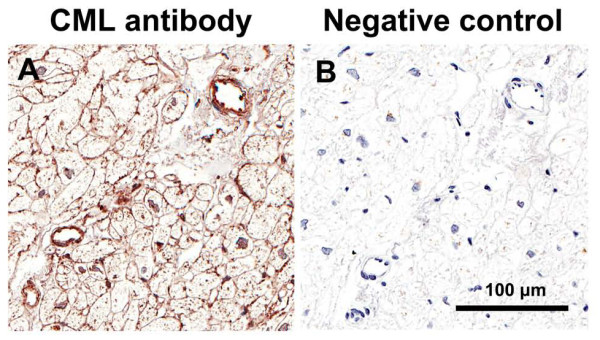
**Immunostaining for Nε-(carboxymethyl)lysine (CML) (A) and negative control section without primary antibody (B), of left ventricular biopsy from control coronary artery bypass graft surgery patient**.

**Figure 9:**
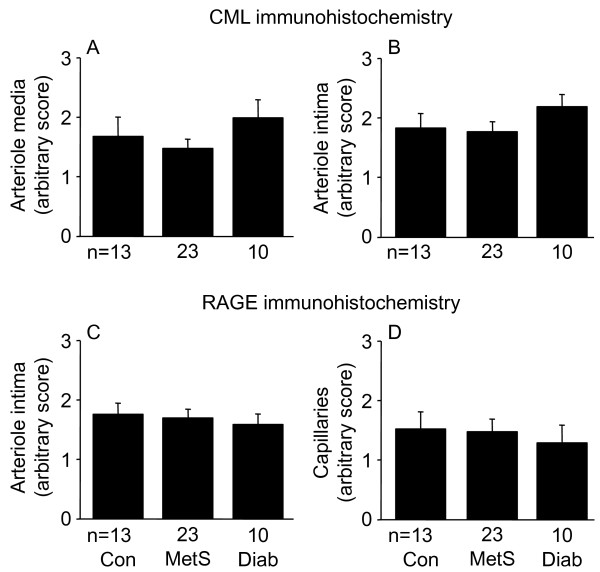
**N^ε^-(carboxymethyl)lysine (CML) in arteriolar media (A) and intima (B), and the receptor for AGEs (RAGE) in arteriolar intima (C) and capillaries (D) determined by immunostaining in left ventricular biopsies from control (Con), metabolic syndrome (MetS), and type 2 diabetic (Diab) men undergoing coronary artery bypass graft surgery**. Data shown as means ± SEM.

**Figure 10:**
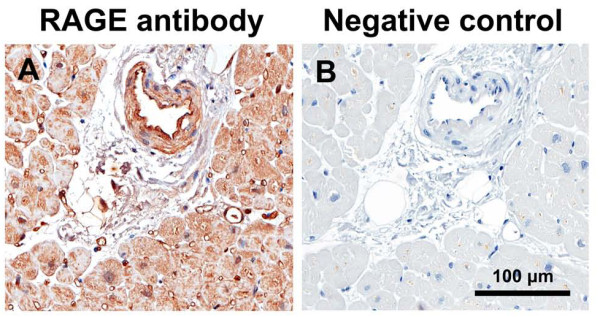
**Immunostaining for the receptor for advanced glycation end-products (RAGE) (A) and negative control section without primary antibody (B), of left ventricular biopsy from control coronary artery bypass graft surgery patient**.

As previously described [33], immunostaining for CML was predominantly localized to the media and intima of arterioles and venules (Figure 8) and we found no difference in CML expression between the three patient groups (Figure 9). RAGE immunostaining was predominantly localized to the intima of arterioles and venules and to capillaries (Figure 10) and we found no difference in RAGE expression between the three patient groups (Figure 9).

### Correlations with duration of diabetes, glycemic control, and insulin resistance

Although the duration of diabetes ranged from 0-30 years, none of the parameters of myocardial structure, microvasculature, and AGE and RAGE expression correlated with diabetes duration in the diabetic subjects. By contrast, diabetes duration correlated with the E/E' ratio (r = 0.87, P = 0.03), with a weaker non-significant correlation with mitral septal E' (r = 0.83, P = 0.08).

Among all patients combined, fasting plasma glucose was positively correlated with the E/E' ratio (r = 0.46, P = 0.02), with a weaker non-significant inverse correlation with mitral septal E' (r = 0.40, P = 0.07), but was not correlated with any of the parameters of myocardial structure, microvasculature, or AGE and RAGE expression. Additionally, neither fasting plasma insulin concentrations nor HOMA2-IR was correlated with any parameter of myocardial structure, microvasculature, or AGE and RAGE expression.

## Discussion

This study investigated the association of type 2 diabetes and the MetS with myocardial fibrosis (assessed by picrosirius red staining and immunostaining for collagen I and III), cardiomyocyte size, capillary length density, diffusion radius, arteriolar dimensions, and myocardial expression of CML and RAGE. We confirmed the impaired diastolic function of type 2 diabetic and MetS patients [2,34], as shown by their increased pulmonary capillary wedge pressure and reduced mitral septal E'. Our data demonstrate that the impaired diastolic function of type 2 diabetic and MetS patients was not dependent on increased myocardial fibrosis, cardiomyocyte hypertrophy, alteration of the myocardial microvascular structure, or increased myocardial expression of CML or RAGE. These data suggest that the increased myocardial fibrosis and CML expression reported by previous studies of diabetic patients with heart failure [3,12,21,22,33] were a consequence, rather than an initiating cause, of impaired cardiac function.

Our findings for myocardial fibrosis and CML expression are in agreement with a recent study by van Heerebeek et al. [22] who studied endomyocardial LV biopsies from patients hospitalized with worsening heart failure. They found no increase in fibrosis or CML expression in LV biopsies of diabetic subjects with normal LV ejection fraction, but increased fibrosis and CML expression in patients with reduced LV ejection fraction [22]. The present study extends these findings to a much earlier stage in the evolution of diabetic cardiomyopathy by study of epicardial LV biopsies from diabetic and MetS patients with increased LV filling pressures without symptomatic heart failure. Although the diabetic patients had reduced mitral septal E', we found no differences between the three patient groups for Doppler measures of left ventricular filling or left atrial size, suggesting that the impairment of diastolic function was much less than that which occurs in heart failure with preserved ejection fraction.

Cardiomyocyte size has been reported to be increased [3,13,17], normal [19], and reduced in diabetic patients [15]. Our finding of no increase in cardiomyocyte width of diabetic and MetS patients is in agreement with reports of similar cardiac mass for diabetic and non-diabetic individuals [35], and the association between increased insulin levels and cardiac hypertrophy is largely accounted for by associations with body size [36]. Moreover, our finding of no effect of diabetes and the MetS on arteriolar dimensions is in agreement with a previous autopsy study of myocardial arteries [20]. Our failure to detect hypertrophy of myocardial arterioles similar to that reported for small arteries in subcutaneous tissue of patients with type 2 diabetes [37] may be because the arterioles examined in our study were much smaller in size, and it is also possible that the effects of diabetes on vascular hypertrophy are different for different vascular beds. The similar capillary length density and arteriolar dimensions of diabetic and non-diabetic patients in this study is in agreement with the similar myocardial perfusion of diabetic and non-diabetic subjects in the fasting state [38,39], although it does not exclude an effect of diabetes and the MetS on microvascular function.

Our finding that RAGE expression was predominantly localized to vascular endothelium and capillaries is in agreement with previous studies [40], although, to our knowledge, this is the first comparison of myocardial RAGE expression in control, diabetic, and MetS patients. Whereas cell-surface-bound RAGE plays an essential role in mediating the effects of AGEs, soluble RAGE may act as an AGE inhibitor, by preventing the binding of AGEs to the cell-surface-bound RAGE receptor [41]. Our finding of similar circulating levels of soluble RAGE and cardiac expression of RAGE suggest RAGE did not participate in the impaired diastolic function of diabetic and MetS patients in our study. The similar plasma CML and LMWF levels of diabetic patients to those of control patients is in agreement with our previous study [42], and likely reflects their well-controlled diabetes.

### Differences between human and animal models of diabetes

Our study highlights possible differences between diabetic humans and animal models of diabetes. In addition to the lack of increased myocardial fibrosis, we found no evidence for the cardiomyocyte and arteriolar hypertrophy and reduced myocardial capillary density reported in animal models of diabetes [7,9-11], which may be related to poorer diabetic control in animal models.

### Alternative mechanisms of impaired diastolic function of diabetes and the MetS

Many mechanisms other than increased fibrosis, cardiomyocyte hypertrophy and alteration in myocardial vasculature have been proposed to explain the diastolic dysfunction of diabetes and the MetS. These include alterations in cardiomyocyte metabolism, abnormal calcium homeostasis and contractile mechanisms, oxidative stress, direct glucotoxicity, and inflammation [1,2,5,8,22,43,44]. Our data are compatible with a role for both insulin resistance and hyperglycemia in the pathogenesis of the diastolic dysfunction of diabetes and the MetS [2].

### Limitations

Our study had a number of limitations. These included a limited sample size because of the need for myocardial biopsies from each patient. However, in a separate study of 11 control, 7 diabetic, and 6 MetS patients with aortic stenosis we similarly found no association of diabetes or the MetS with increased myocardial fibrosis or altered cardiomyocyte width, capillary length density, diffusion radius, or arteriolar dimensions, although, in comparison with the patients in this study, aortic stenosis patients had more interstitial fibrosis, greater cardiomyocyte width, lower capillary length density, and greater diffusion radius (unpublished data). Another limitation was the inherent selection bias caused by the sampling of patients presenting for coronary artery bypass graft surgery. However, in a comparison of aortic stenosis patients with and without coronary artery disease we found no effect of coronary artery disease on myocardial fibrosis, cardiomyocyte width, capillary length density, diffusion radius, or arteriolar dimensions (unpublished data). Patients with coronary artery disease were an important patient group to study because of the high prevalence of coronary artery disease in diabetic patients [45,46], and coronary artery disease patients with diabetes are twice as likely as those without diabetes to develop heart failure [6]. Although the frequent use of antihypertensive therapy, including inhibitors of the renin angiotensin system and statin therapy, may have attenuated the effect of MetS and diabetes on the myocardium, the MetS and diabetic patients in this study had evidence of diastolic dysfunction. Moreover, diabetes remains a predictor of heart failure when these therapies are included in multivariate analysis [6]. To avoid the effect of coronary stenoses on myocardial structure and the microvasculature we took particular care to collect biopsies from the same epicardial region of the LV myocardium without evidence of ischemia or wall motion abnormality, which was proximal to significant flow-limiting coronary stenoses and collaterals. However, we do not know if the data obtained apply to other regions of the myocardium, such as myocardium distal to significant flow-limiting coronary stenoses. It is also unknown whether our findings are relevant to the right ventricle, which may also contribute to diabetic cardiomyopathy [47]. Moreover, the generalizability of our findings is limited by the exclusion of women. Although we had echocardiographic data for only a limited number of patients we had hemodynamic data, including ventriculograms and pulmonary capillary wedge pressures, which provided a direct measure of LV filling pressure, for all patients.

## Conclusions

We compared MetS (pre-diabetic) and diabetic patients with patients without these conditions in order to examine the effects of the insulin-resistant state before diabetes (and anti-diabetes medication) commenced, and the effects of diabetes before heart failure developed. We found that the increased LV filling pressures of MetS and type 2 diabetic patients were not dependent on increased myocardial fibrosis or alteration in cardiomyocyte size, capillary length density, diffusion radius, arteriolar dimensions, or myocardial expression of CML and RAGE. Our findings suggest that the increased myocardial fibrosis and CML expression, cardiomyocyte hypertrophy, and altered microvasculature structure previously described in diabetic heart disease were a consequence, rather than an initiating cause, of cardiac dysfunction.

## Abbreviations

AGEs: advanced glycation end-products; ANOVA: analysis of variance; ARB: angiotensin receptor blocker; CML: Nε-(carboxymethyl)lysine; eGFR: estimated glomerular filtration rate calculated using the Modification of Diet in Renal Disease study equation; HOMA: Homeostasis Model Assessment calculator version 2.2; LMWF: low molecular weight fluorophore; LV: left ventricle; MetS: metabolic syndrome; NT-proBNP: amino-terminal-pro-B-type natriuretic peptide; RAGE: receptor for AGEs.

## Competing interests

The authors declare that they have no competing interests.

## Authors' contributions

DJC, JBS, AJJ, DLP, MY, JFK, AEN, CGS, MJB, and DJK participated in the design of the study and acquisition of the data. DJC, JBS, DLP, MJB, and DJK participated in the analysis and interpretation of data. DJC participated in manuscript preparation. JBS, AJJ, DLP, MY, JFK, AEN, CGS, MJB, and DJK participated in review of the manuscript. All authors have read and approved the final manuscript.

## References

[B1] Fang ZY Prins JB Marwick TH Diabetic cardiomyopathy: evidence, mechanisms, and therapeutic implications Endocr Rev 2004 25 543 567 10.1210/er.2003-0012 15294881

[B2] Horwich TB Fonarow GC Glucose, obesity, metabolic syndrome, and diabetes relevance to incidence of heart failure J Am Coll Cardiol 2010 55 283 293 10.1016/j.jacc.2009.07.029 20117431 PMC2834416

[B3] Rubler S Dlugash J Yuceoglu YZ Kumral T Branwood AW Grishman A New type of cardiomyopathy associated with diabetic glomerulosclerosis Am J Cardiol 1972 30 595 602 10.1016/0002-9149(72)90595-4 4263660

[B4] Kannel WB Hjortland M Castelli WP Role of diabetes in congestive heart failure: the Framingham study Am J Cardiol 1974 34 29 34 10.1016/0002-9149(74)90089-7 4835750

[B5] Poornima IG Parikh P Shannon RP Diabetic cardiomyopathy: the search for a unifying hypothesis Circ Res 2006 98 596 605 10.1161/01.RES.0000207406.94146.c2 16543510

[B6] Lewis EF Solomon SD Jablonski KA Rice MM Clemenza F Hsia J Maggioni AP Zabalgoitia M Huynh T Cuddy TE Gersh BJ Rouleau J Braunwald E Pfeffer MA Predictors of heart failure in patients with stable coronary artery disease: a PEACE study Circ Heart Fail 2009 2 209 216 10.1161/CIRCHEARTFAILURE.108.820696 19808342 PMC3009573

[B7] Candido R Forbes JM Thomas MC Thallas V Dean RG Burns WC Tikellis C Ritchie RH Twigg SM Cooper ME Burrell LM A breaker of advanced glycation end products attenuates diabetes-induced myocardial structural changes Circ Res 2003 92 785 792 10.1161/01.RES.0000065620.39919.20 12623881

[B8] Hsueh W Abel ED Breslow JL Maeda N Davis RC Fisher EA Dansky H McClain DA McIndoe R Wassef MK Rabadan-Diehl C Goldberg IJ Recipes for creating animal models of diabetic cardiovascular disease Circ Res 2007 100 1415 1427 10.1161/01.RES.0000266449.37396.1f 17525381

[B9] Connelly KA Kelly DJ Zhang Y Prior DL Advani A Cox AJ Thai K Krum H Gilbert RE Inhibition of protein kinase C-beta by ruboxistaurin preserves cardiac function and reduces extracellular matrix production in diabetic cardiomyopathy Circ Heart Fail 2009 2 129 137 10.1161/CIRCHEARTFAILURE.108.765750 19808328

[B10] Hayashi T Sohmiya K Ukimura A Endoh S Mori T Shimomura H Okabe M Terasaki F Kitaura Y Angiotensin II receptor blockade prevents microangiopathy and preserves diastolic function in the diabetic rat heart Heart 2003 89 1236 1242 10.1136/heart.89.10.1236 12975429 PMC1767873

[B11] Gross ML Heiss N Weckbach M Hansen A El-Shakmak A Szabo A Munter K Ritz E Amann K ACE-inhibition is superior to endothelin A receptor blockade in preventing abnormal capillary supply and fibrosis of the heart in experimental diabetes Diabetologia 2004 47 316 324 10.1007/s00125-003-1309-z 14727024

[B12] Fein FS Sonnenblick EH Diabetic cardiomyopathy Prog Cardiovasc Dis 1985 27 255 270 10.1016/0033-0620(85)90009-X 3880919

[B13] Nunoda S Genda A Sugihara N Nakayama A Mizuno S Takeda R Quantitative approach to the histopathology of the biopsied right ventricular myocardium in patients with diabetes mellitus Heart Vessels 1985 1 43 47 10.1007/BF02066486 4093355

[B14] Shimizu M Umeda K Sugihara N Yoshio H Ino H Takeda R Okada Y Nakanishi I Collagen remodelling in myocardia of patients with diabetes J Clin Pathol 1993 46 32 36 10.1136/jcp.46.1.32 7679418 PMC501107

[B15] Kawaguchi M Techigawara M Ishihata T Asakura T Saito F Maehara K Maruyama Y A comparison of ultrastructural changes on endomyocardial biopsy specimens obtained from patients with diabetes mellitus with and without hypertension Heart Vessels 1997 12 267 274 10.1007/BF02766802 9860193

[B16] van Hoeven KH Factor SM A comparison of the pathological spectrum of hypertensive, diabetic, and hypertensive-diabetic heart disease Circulation 1990 82 848 855 10.1161/01.CIR.82.3.848 2394006

[B17] Fischer VW Barner HB Leskiw ML Capillary basal laminar thichness in diabetic human myocardium Diabetes 1979 28 713 719 10.2337/diabetes.28.8.713 446928

[B18] Factor SM Minase T Sonnenblick EH Clinical and morphological features of human hypertensive-diabetic cardiomyopathy Am Heart J 1980 99 446 458 10.1016/0002-8703(80)90379-8 6444776

[B19] Kuethe F Sigusch HH Bornstein SR Hilbig K Kamvissi V Figulla HR Apoptosis in patients with dilated cardiomyopathy and diabetes: a feature of diabetic cardiomyopathy? Horm Metab Res 2007 39 672 676 10.1055/s-2007-985823 17846975

[B20] Sunni S Bishop SP Kent SP Geer JC Diabetic cardiomyopathy. A morphological study of intramyocardial arteries Arch Pathol Lab Med 1986 110 375 381 3754419

[B21] Frustaci A Kajstura J Chimenti C Jakoniuk I Leri A Maseri A Nadal-Ginard B Anversa P Myocardial cell death in human diabetes Circ Res 2000 87 1123 1132 11110769 10.1161/01.res.87.12.1123

[B22] van Heerebeek L Hamdani N Handoko ML Falcao-Pires I Musters RJ Kupreishvili K Ijsselmuiden AJ Schalkwijk CG Bronzwaer JG Diamant M Borbely A van der Velden J Stienen GJ Laarman GJ Niessen HW Paulus WJ Diastolic stiffness of the failing diabetic heart: importance of fibrosis, advanced glycation end products, and myocyte resting tension Circulation 2008 117 43 51 10.1161/CIRCULATIONAHA.107.728550 18071071

[B23] Sutherland CG Fisher BM Frier BM Dargie HJ More IA Lindop GB Endomyocardial biopsy pathology in insulin-dependent diabetic patients with abnormal ventricular function Histopathology 1989 14 593 602 10.1111/j.1365-2559.1989.tb02200.x 2759556

[B24] Campbell DJ Somaratne JB Jenkins AJ Prior DL Yii M Kenny JF Newcomb AE Kelly DJ Black MJ Differences in myocardial structure and coronary microvasculature between men and women with coronary artery disease Hypertension 2011 57 186 192 10.1161/HYPERTENSIONAHA.110.165043 21135353

[B25] Alberti KG Zimmet P Shaw J Metabolic syndrome--a new world-wide definition. A Consensus Statement from the International Diabetes Federation Diabet Med 2006 23 469 480 10.1111/j.1464-5491.2006.01858.x 16681555

[B26] Zhu S Heshka S Wang Z Shen W Allison DB Ross R Heymsfield SB Combination of BMI and Waist Circumference for Identifying Cardiovascular Risk Factors in Whites Obes Res 2004 12 633 645 10.1038/oby.2004.73 15090631

[B27] The Expert Committee on the Diagnosis and Classification of Diabetes Mellitus Report of the expert committee on the diagnosis and classification of diabetes mellitus Diabetes Care 2003 26 Suppl 1 S5 S20 12502614 10.2337/diacare.26.2007.s5

[B28] Levey AS Bosch JP Lewis JB Greene T Rogers N Roth D A more accurate method to estimate glomerular filtration rate from serum creatinine: a new prediction equation. Modification of Diet in Renal Disease Study Group Ann Intern Med 1999 130 461 470 10075613 10.7326/0003-4819-130-6-199903160-00002

[B29] Wallace TM Levy JC Matthews DR Use and abuse of HOMA modeling Diabetes Care 2004 27 1487 1495 10.2337/diacare.27.6.1487 15161807

[B30] Januszewski AS Thomas MC Chung SJ Karschimkus CS Rowley KG Nelson C O'Neal D Wang Z Best JD Jenkins AJ Plasma low-molecular weight fluorescence in type 1 diabetes mellitus Ann NY Acad Sci 2005 1043 655 661 10.1196/annals.1333.074 16037289

[B31] Tomita H Egashira K Ohara Y Takemoto M Koyanagi M Katoh M Yamamoto H Tamaki K Shimokawa H Takeshita A Early induction of transforming growth factor-β via angiotensin II type 1 receptors contributes to cardiac fibrosis induced by long-term blockade of nitric oxide synthesis in rats Hypertension 1998 32 273 279 9719054 10.1161/01.hyp.32.2.273

[B32] Gordon H Sweets HH A simple method for the silver impregnation of reticulin Am J Pathol 1936 12 545 552 19970284 PMC1911089

[B33] Schalkwijk CG Baidoshvili A Stehouwer CD van Hinsbergh VW Niessen HW Increased accumulation of the glycoxidation product N^ε^-(carboxymethyl)lysine in hearts of diabetic patients: generation and characterisation of a monoclonal anti-CML antibody Biochim Biophys Acta 2004 1636 82 89 15164755 10.1016/j.bbalip.2003.07.002

[B34] Page A Dumesnil JG Clavel MA Chan KL Teo KK Tam JW Mathieu P Despres JP Pibarot P Metabolic syndrome is associated with more pronounced impairment of left ventricle geometry and function in patients with calcific aortic stenosis: a substudy of the ASTRONOMER (Aortic Stenosis Progression Observation Measuring Effects of Rosuvastatin) J Am Coll Cardiol 2010 55 1867 1874 10.1016/j.jacc.2009.11.083 20413039

[B35] Rijzewijk LJ van der Meer RW Smit JW Diamant M Bax JJ Hammer S Romijn JA de Roos A Lamb HJ Myocardial steatosis is an independent predictor of diastolic dysfunction in type 2 diabetes mellitus J Am Coll Cardiol 2008 52 1793 1799 10.1016/j.jacc.2008.07.062 19022158

[B36] Ilercil A Devereux RB Roman MJ Paranicas M O'Grady MJ Lee ET Welty TK Fabsitz RR Howard BV Associations of insulin levels with left ventricular structure and function in American Indians: the strong heart study Diabetes 2002 51 1543 1547 10.2337/diabetes.51.5.1543 11978654

[B37] Schofield I Malik R Izzard A Austin C Heagerty A Vascular structural and functional changes in type 2 diabetes mellitus: evidence for the roles of abnormal myogenic responsiveness and dyslipidemia Circulation 2002 106 3037 3043 10.1161/01.CIR.0000041432.80615.A5 12473548

[B38] Scognamiglio R Negut C De Kreutzenberg SV Tiengo A Avogaro A Postprandial myocardial perfusion in healthy subjects and in type 2 diabetic patients Circulation 2005 112 179 184 10.1161/CIRCULATIONAHA.104.495127 15998667

[B39] Rijzewijk LJ van der Meer RW Lamb HJ de Jong HW Lubberink M Romijn JA Bax JJ de Roos A Twisk JW Heine RJ Lammertsma AA Smit JW Diamant M Altered myocardial substrate metabolism and decreased diastolic function in nonischemic human diabetic cardiomyopathy: studies with cardiac positron emission tomography and magnetic resonance imaging J Am Coll Cardiol 2009 54 1524 1532 10.1016/j.jacc.2009.04.074 19815124

[B40] Ritthaler U Deng Y Zhang Y Greten J Abel M Sido B Allenberg J Otto G Roth H Bierhaus A Expression of receptors for advanced glycation end products in peripheral occlusive vascular disease Am J Pathol 1995 146 688 694 7887450 PMC1869189

[B41] Barlovic DP Soro-Paavonen A Jandeleit-Dahm KA RAGE biology, atherosclerosis and diabetes Clin Sci (Lond) 2011 121 43 55 10.1042/CS20100501 21457145

[B42] Campbell DJ Kladis A Zhang Y Jenkins AJ Prior DL Yii M Kenny JF Black MJ Kelly DJ Increased tissue kallikrein levels in type 2 diabetes Diabetologia 2010 53 779 785 10.1007/s00125-009-1645-8 20225398

[B43] Diamant M Lamb HJ Groeneveld Y Endert EL Smit JW Bax JJ Romijn JA de Roos A Radder JK Diastolic dysfunction is associated with altered myocardial metabolism in asymptomatic normotensive patients with well-controlled type 2 diabetes mellitus J Am Coll Cardiol 2003 42 328 335 10.1016/S0735-1097(03)00625-9 12875772

[B44] Schrauwen-Hinderling VB Kooi ME Hesselink MK Jeneson JA Backes WH van Echteld CJ van Engelshoven JM Mensink M Schrauwen P Impaired in vivo mitochondrial function but similar intramyocellular lipid content in patients with type 2 diabetes mellitus and BMI-matched control subjects Diabetologia 2007 50 113 120 17093944 10.1007/s00125-006-0475-1

[B45] Zeina AR Odeh M Rosenschein U Zaid G Barmeir E Coronary artery disease among asymptomatic diabetic and nondiabetic patients undergoing coronary computed tomography angiography Coron Artery Dis 2008 19 37 41 10.1097/MCA.0b013e3282f2f19e 18281814

[B46] Rivera JJ Nasir K Choi EK Yoon YE Chun EJ Choi SI Choi DJ Brancati FL Blumenthal RS Chang HJ Detection of occult coronary artery disease in asymptomatic individuals with diabetes mellitus using non-invasive cardiac angiography Atherosclerosis 2009 203 442 448 10.1016/j.atherosclerosis.2008.07.030 18822414

[B47] van den Brom CE Bosmans JW Vlasblom R Handoko LM Huisman MC Lubberink M Molthoff CF Lammertsma AA Ouwens MD Diamant M Boer C Diabetic cardiomyopathy in Zucker diabetic fatty rats: the forgotten right ventricle Cardiovasc Diabetol 2010 9 25 10.1186/1475-2840-9-25 20550678 PMC2898761

[B48] Rentrop KP Cohen M Blanke H Phillips RA Changes in collateral channel filling immediately after controlled coronary artery occlusion by an angioplasty balloon in human subjects J Am Coll Cardiol 1985 5 587 592 10.1016/S0735-1097(85)80380-6 3156171

